# Preparation, Characterization, and Properties of UV-Curable Coating Doped with Nano-SiO_2_

**DOI:** 10.3390/ma16247576

**Published:** 2023-12-09

**Authors:** Tianlei Chen, Rong Zhong, Zhengjie Wang

**Affiliations:** Department of Materials Chemistry, School of Environmental and Chemical Engineering, Nanchang Hangkong University, Nanchang 330063, China; 2102077600029@stu.nchu.edu.cn (T.C.); 1902077600105@stu.nchu.edu.cn (Z.W.)

**Keywords:** UV-curable coating, nano-SiO_2_ modification, high-energy ball milling, organic–inorganic composite coating

## Abstract

In this study, a hydrophobic, wear-resistant ultraviolet (UV)-curable coating was investigated as an alternative to traditional coatings with low hardness and high susceptibility to scratching. The SiO_2_ nanoparticles were ground and modified using high-energy ball milling, during which the surface energy of nano-SiO_2_ particles rapidly increased as their particle size decreased. Different proportions of modified nano-SiO_2_ particles were added to the coating and cured into a film. The structure of the composite coating was analyzed via infrared spectroscopy, scanning electron microscopy, and X-ray diffraction, which confirmed the successful preparation of the composite coating. The mechanical and optical property tests of the coating were investigated. With a 5% nano-SiO_2_ content, the hardness of the coating reached 5H, whereas the adhesion was poor (2B), and the flexibility was 1. The overall comprehensive performance of the coating was best when the addition amount was 3%. The coating exhibited good hardness, flexibility, and adhesion. The hardness of the coating reached 4H, the adhesion was 4B, the flexibility was 5, the coating haze was 12.38 HZ, and the contact angle was 118°.

## 1. Introduction

UV-curable coatings are generally composed of UV-curable resins, active diluents (monomers), photoinitiators, and small amounts of additives [1,2,3,4], typically requiring short periods of UV irradiation to affect curing [5]. With the development of science and technology, plastics have become an indispensable material in the production of flexible products; however, their application in the coatings field is limited, owing to their low surface hardness and poor wear resistance [6]. Therefore, to improve these properties, the hardening method of UV-curable coatings on the surface of plastics has been considered because of its low cost, convenience, and simplicity [7,8,9,10]. In some cases, pure organic coatings have good compatibility, adhesion, and flexibility with the substrate after curing, owing to their molecular structural characteristics [11,12,13,14]. However, owing to their inherent characteristics, organic coatings often exhibit high shrinkage [15], poor wear resistance [16], and low hardness [17] after curing. Inorganic films mainly comprise inorganic oxide and non-oxide materials [18,19], which exist in crystalline and amorphous forms and are mainly deposited on plastic substrates using PVD [20] and CVD [21] technology. Plastic deformation or softening is often caused by the high temperature of the deposited plastic substrate [22]. In addition, the bonding between inorganic films and plastic substrates is poor, and they easily peel off [23,24]. Organic/inorganic hybrid films attempt to integrate the advantages of the aforementioned films and overcome their shortcomings, which is a current research hotspot. Currently, most transparent hard organic/inorganic film materials are based on polysiloxanes or other inorganic polymers. This hybrid material has an O–M–O (M = Si, Ti, Al, Zr, or other metal ions) network structure as the basic skeleton, and its adhesion to plastic substrates is improved by adding organic components [25,26,27]. After more than ten years of development, the synthesis methods for hybrid materials have continuously improved. Si-containing organic/inorganic nanohybrid materials are an important class of hybrid materials. These materials are mainly synthesized using methods such as sol–gel [28], in situ polymerization [29], intercalation [30], self-assembly [31], and blending [32].

The small particle size, insufficient atomic coordination, large specific surface area, and high surface energy of nano-SiO_2_ particles contribute to their high activity, instability, and easy agglomeration [33,34]. Appropriate physical or chemical methods [35,36,37,38] must be adopted to obtain nano-SiO_2_ particles with good dispersion, a small particle size, and narrow particle size distribution and to enhance their compatibility with organic systems. High-energy ball milling is a surface modification technology that significantly reduces reaction activation energy, refines grains, and improves the powder activity and uniformity of particle distribution. Furthermore, the method improves the combination of the interface between the SiO_2_ and the resin, promotes the diffusion of solid ions, and induces low-temperature chemical reactions, thus improving the compactness and electrical, thermal, and other material properties. This method is also an efficient material preparation technology.

The high-energy ball-milling method is a better method for the preparation of nanoparticles than the blending method and ultrasonic method in our experiments. To achieve high hydrophobicity and scratch resistance, inorganic and organic hybrid UV-curable coatings containing nano-SiO_2_ have become a research subject. In our previous study, inorganic and organic hybrid UV-curable coatings for different nano-metal oxides were investigated [39,40]. In this paper, the high-energy ball-milling method was used to crush and grind nanoparticles, which were then modified using a dispersant. The modified nano-SiO_2_ particles were added to a UV-coating system to prepare an organic–inorganic composite coating doped with nano-SiO_2_ particles. The experimental formula was optimized by adjusting the type and quantity of monomers and the proportion of resin. The mechanical properties, such as wear resistance, hardness, and adhesion, of the composite coating with different nano-SiO_2_ contents were explored. The particle size of the nano-SiO_2_ particles was measured and analyzed using a nanoparticle size meter, and their dispersion in the coating was examined via scanning electron microscopy (SEM). The coating structure was analyzed via Fourier transform infrared spectrometry (FTIR) and X-ray diffraction. The thermal stability of the coating with different SiO_2_ contents was tested using a thermogravimetric analyzer, while its hydrophobicity was tested using a contact angle meter.

## 2. Materials and Methods

### 2.1. Chemicals and Reagents

Nano-SiO_2_ powder (99.99%) was purchased from Aladdin Chemical Co., Ltd. (Shanghai, China). Afcona 4071 (44–46 wt%) was purchased from Evkona Additives Co., Ltd. (Haimeng, China). Ethyl acetate, toluene, and n-butyl acetate were purchased from Guangzhou Xinguan Chemical Co., Ltd. (Guangzhou, China). Polyurethane acrylate (B-619 W) was purchased from Guangzhou Boxing Chemical Co., Ltd. (Guangzhou, China). The monomers hexanedioldiacrylate (HDDA) and acrylated dipentaerythritol (DPHA) were purchased from Taiwan Changxing Chemical Co., Ltd. (Guangzhou, China). Photoinitiator (184) was purchased from Tianjin JiuriNew Materials Co., Ltd. (Tianjin, China). PET film was purchased from Shenzhen Puti Technology Co., Ltd. (Shenzhen, China).

### 2.2. Modification of Nano-SiO_2_ via High-Energy Ball Milling

**Scheme 1:**
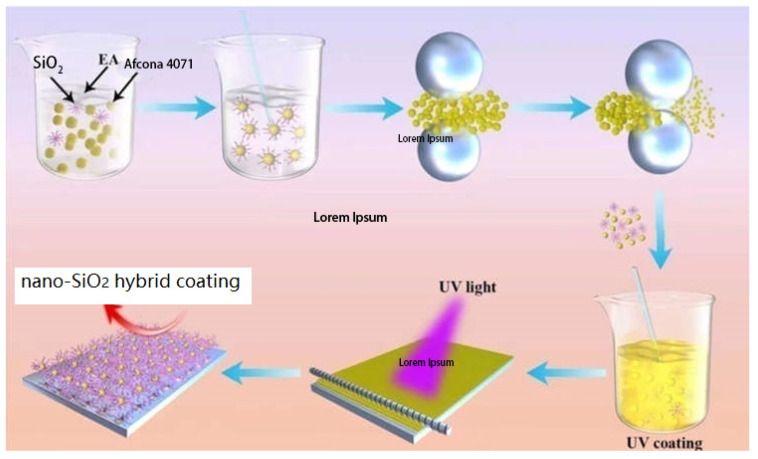
Preparation process of composite coatings.

Dispersions were prepared from the as-purchased SiO_2_, the polyurethane dispersant Afcona 4071 (a 44–46% solid solution), and ethyl acetate as solvent. The dispersions were designed to have a final solid content of 50%, and the dispersions contained 0 (the control, without ethyl acetate addition), 1%, 2%, 3%, 4%, and 5% by weight of SiO_2_ powder, with re-adjustment of total solid content via ethyl acetate addition. In turn, each dispersion was transferred to the feed chamber of a horizontal bead mill loaded with 0.1 mm zirconia (140 mL) beads and milled for 1 h at a speed of 3800 rpm. The volume of the ball-mill container was 200 mL. The ratios of sample:balls:container were x:140 mL:200 mL. The sample was cyclically injected into the ball-mill container via a pump. The product dispersions were collected and used in the coating formulations. The preparation process of composite coatings is presented in Scheme 1.

### 2.3. Preparation of Composite Coatings

B-619W was used as the main resin, HDDA and DPHA were used as the active diluents, 184 was used as the photoinitiator, and ethyl acetate was used as the solvent. The resin, monomers, and photoinitiator were weighed in appropriate proportions and mixed until an even consistency was obtained. Subsequently, 1–5% of nano-SiO_2_ dispersion was added, following which the evenly mixed UV-curable coating was diluted until an optimal coating consistency was obtained. A wire rod was used to spread the coating onto the 18.8 mm thick PET substrate, which was then baked in an oven for 1 min at 80 °C. The dried coating was cured in a UV-curing machine prior to the evaluation of its performance. An RX300-1 UV curing machine (Ergu Photoelectric Co., Ltd., Dongguan, China) was used. The main wavelength of the mercury light was 365 nm. The irradiation distance was about 15 cm. The curing time of the sample was less than 60 s at room temperature.

### 2.4. Characterization

#### 2.4.1. FTIR Spectra

The cured film was peeled off from the PET substrate and ground into a powder. FTIR spectra of the sample were generated via an American Nicolet Magna380 infrared spectrometer (Waltham, MA, USA), using the KBr compression method, with a resolution of 4 cm^−1^ and a limited scanning wavelength range of 400–4000 cm^−1^. The absorption of the coating was detected in this band.

#### 2.4.2. XRD Spectra

The cured film was peeled off from the glass slide, ground into a powder, and analyzed using a D8 Advance (Bruker, Saarbrücken, SAAR, Germany) X-ray powder diffractometer. Bruker D8 Advance diffractometer was operated at 40 kV and 40 mA with a CuKα radiation source in reflection mode using a two-circle goniometer in a Bragg–Brentano arrangement. The scanning range was 20~70°, and the scanning speed was 5°/min.

#### 2.4.3. Scanning Electron Microscope (SEM) Analysis

A small amount of the sample was uniformly coated on the silicon wafer with a rubber-tipped dropper and cured into a film. Prior to electron imaging, samples were coated with 10 nm of gold using a Leica ACE600 sputter coater (Leica Microsystems Inc., Deerfield, IL, USA). The sample was put into a NovaSEM450 field emission scanning electron microscope (FEI Company, Hillsboro, OR, USA) for imaging.

#### 2.4.4. Thermogravimetric Analysis

A TG-209 thermogravimetric (Netzsch, Selb, BW, Germany) analyzer was used to weigh the quantitatively dried samples, which were placed into a crucible for testing in a nitrogen atmosphere. The test temperature range was 0~800 °C, the heating rate was 10 °C/min, and the air flow rate was 100 mL/min. The data were recorded every 0.5 s.

#### 2.4.5. Mechanical Property Tests

The pencil hardness test was conducted according to the ASTM D3363-05 standard [41]. The adhesion refers to ASTMD3002 (0B–5B). Portable pencil hardness tester (QHQ-A, Dongguan Guohua Experimental Instrument Co., Ltd., Dongguan, China) was used to test the hardness of the coating. A pencil was scratched five times on the film under a certain applied force (500 g or 750 g), and the hardness of the paint film was determined via the pencil hardness that produced no scratch. A paint film elasticity tester (QTX, Tianjin Hongjuli Experimental Instrument Factory, Tianjin, China) was used to test the flexibility of the coating according to the GB/T 1731-1993 standard [42].

#### 2.4.6. Haze Tests

Transmittance haze tester (JZ-WGT-S) was used. Haze means the ratio of the scattered light flux to the transmitted light flux that passes through the specimen and deviates from the direction of the incident light. Haze is an important parameter for the optical transparency of transparent or semi-transparent coatings. After the calibration of the machine, the cured film was placed in a haze meter, and haze testing was performed on it. Each coating was measured three times, taking the average value as the final haze, usually expressed in Hz.

#### 2.4.7. Wear Resistance Test

Steel velvet wear resistance testing machine (339-GSR, Shenzhen, China) was used to test the wear resistance properties. The cut steel wool was wrapped around the friction head. Then, the diaphragm was fixed in the friction area as flat as possible to prevent it from loosening during friction. Various loads, such as 500 g and 1000 g, and the frequency of friction cycles were applied. The friction head was rubbed back and forth. After the friction test, the surface of the diaphragm was observed.

#### 2.4.8. The Contact Angle Test

A water droplet angle tester and the computer connected to it were turned on, and 1–2 drops were added on the coating with 1 μL water droplets and, after being dropped, were connected to the water droplet angle through the rising sample stage. After clicking on the freeze button, a measuring ruler tangent to the edge of the droplet was made by clicking on the shortcut button, and then, the value of the contact angle was recorded.

#### 2.4.9. Particle Size Analysis

Zhenli Optical Laser Particle Sizer (Truth Optical Instrument Co., Ltd., Zhuhai, China) was used. A certain amount of nanodispersion was sucked into a beaker using a pipette, diluting it 20–30 times with toluene, stirring evenly. Then, a pipette was used to absorb the diluted dispersion and add it to a colorimetric dish. Afterward, the dispersion on the colorimetric dish was placed into a particle size analyzer to test the particle size. Each sample was measured in 3 groups, with each group measured 10 times. D10, D50, and D90 were used for analyzing particle sizes. D10 represents the particle size of a sample when the cumulative particle size distribution reaches 10%. This means that 10% of the particles are smaller than it. D50 represents the particle size of a sample when the cumulative particle size distribution percentage reaches 50%. This means particles larger than it accounted for 50%, while smaller particles also accounted for 50%. D50 is often used to represent the average particle size of powders. D90 represents the particle size of a sample when the cumulative particle size distribution reaches 90%. This means that 90% of the particles are smaller than it.

## 3. Results

### 3.1. Effect of Different Afcona 4071 Additions on the Particle Size of Nano-SiO_2_

**Figure 1:**
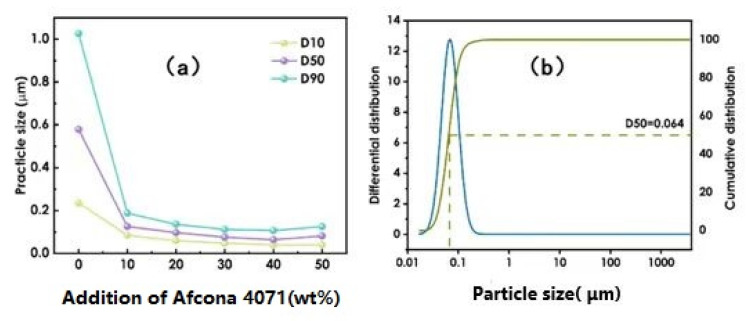
(**a**) Effect of different dispersion additions on size of nano-SiO_2_ particles, (**b**) the distribution of SiO_2_ when Afcona 4071 was 40 wt%.

Figure 1a,b show the effect of different concentrations of dispersant on the size of nano-SiO_2_ particles and their particle size distribution when Afcona 4071 was added, respectively. The used Afcona 4071:SiO_2_ weight ratios are 0:100, 10:90, 20:80, 30:70, 40:60, and 50:50 wt%. As shown in Figure 1a, the initial average particle size of nano-SiO_2_ was 579 nm. After 1 h of high-speed dispersion, the average particle sizes of the SiO_2_ were 126, 97, 76, 64, and 82 nm for different SiO_2_ contents. When only a small amount of dispersant is added, the organic groups are not sufficient to coat the nano-SiO_2_ particles completely. Dispersion is, thus, hindered by an increase in agglomeration caused by covalent bonds and/or van der Waals forces. Our findings demonstrated that an optimal dispersion was achieved with 40% Afcona 4071, with the size of nano-SiO_2_ particles at their smallest. As shown in Figure 1b, after grinding and dispersion, the average particle size was 64 nm, with a narrow particle size distribution and uniform particle size.

### 3.2. Coating Formulation Optimization and Coating Performance Test

(1) Effect of monomer type and dosage on coating properties

In Table 1, the main resin (B-619W) and photoinitiator 184 were kept at 80% and 5%, respectively. The HDDA:DPHA ratios were 1:0, 1:1, 1:2, 2:1, and 0:1. Coating preparation, application, and testing were carried out according to the methods described in Section 2.3. Coating properties, as listed in Table 2, were obtained at a coating thickness of 4 μm. These properties indicate that an increase in the proportion of DPHA improved the hardness of the coating, whereas the adhesion and flexibility of the coating decreased. With increasing proportions of HDDA, the adhesion and flexibility of the coating improved, whereas the hardness of the coating was low. The overall performance of the coating was optimal at a 1:1 ratio of HDDA:DPHA, with an adhesion of 4B, a flexibility of 6, and a hardness of 2H/750 g.

(2) Effect of different resin ratios on coating properties

In Table 3, the ratio of the monomers (HDDA:DPHA) was maintained at 1:1, the proportion of the photoinitiator was maintained at 5%, and the proportions of the resin were varied. Coating preparation, application, and testing were carried out according to the methods described in Section 2.3. The coating properties, as listed in Table 4, were obtained at a coating thickness of 4 μm. The change in the coating performance was nonlinear. An increase in resin content led to a higher degree of crosslinking, which resulted in the improved hardness and adhesion of the coating. Since B-619W is a hexahedral resin, the coating hardness is expected to decrease with increased proportions of resin. Our findings confirmed good adhesion and flexibility, as well as a hardness that reached 2H/750 g when the proportion of resin was 75%.

### 3.3. IR Testing of Composite Coatings

**Figure 2:**
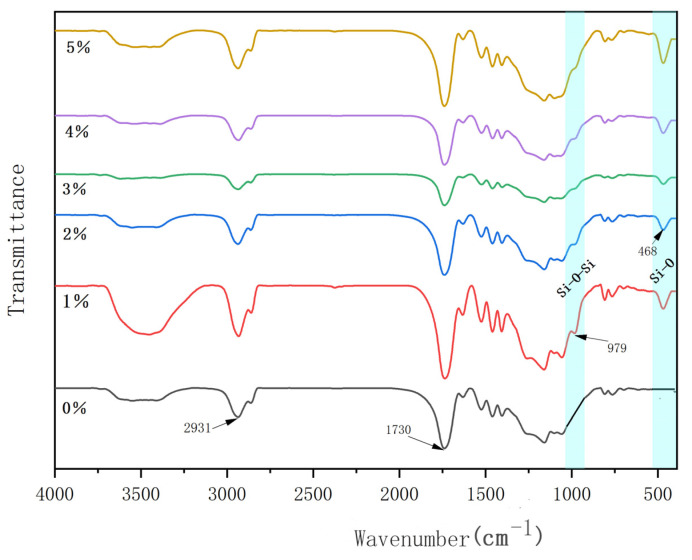
FTIR spectra of coatings with different contents of nano-SiO_2_.

Figure 2 shows the infrared spectra of coatings that contain varying contents of nano-SiO_2_, i.e., 0%, 1%, 2%, 3%, 4%, and 5%. A characteristic vibration peak of C=O is seen at 1730 cm^−1^. The wide band centered around 3500 cm^−1^ is attributed to the O–H stretching vibration. For the sample with 1% nano-SiO_2_ content, it could contain some residual H_2_O. The absorption peak at 2931 cm^−1^ may either be attributed to the characteristic absorption peak of –CH_2_ present in the mixture or to the O–H bending vibration caused by an incomplete group reaction. The spectra of the coatings that contained SiO_2_ not only retained the characteristic peak seen in the coating without SiO_2_ but also showed the emergence of an additional absorption peak at 468 cm^−1^, caused by the bending vibration of the Si–O bond. The absorption peak at 979 cm^−1^ can be attributed to the antisymmetric stretching vibration of the Si–O–Si bonds [43]. This proves that the composite coating formed a Si–O–Si network structure.

### 3.4. SEM Analysis of Composite Coatings

**Figure 3:**
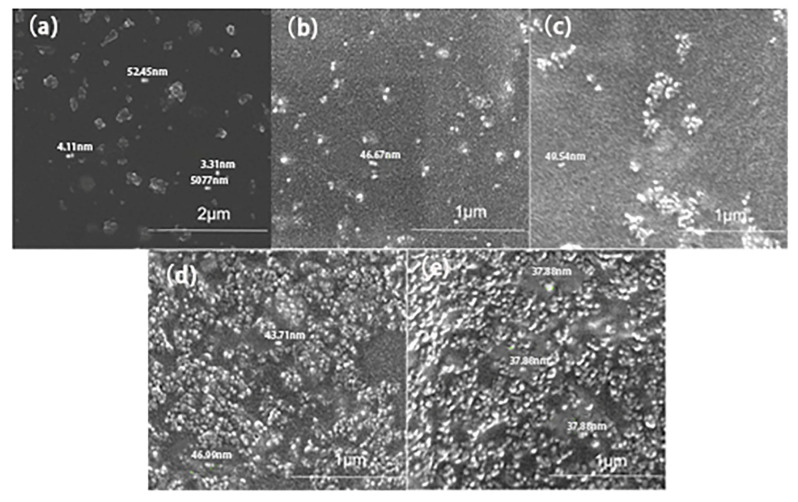
SEM of composite coatings with (**a**) 1%, (**b**) 2%, (**c**) 3%, (**d**) 4%, and (**e**) 5% nano-SiO_2_.

Figure 3 shows the structure and surface morphology of the coating with 1%, 2%, 3%, 4%, and 5% amounts of nano-SiO_2_ particles. It is clear from Figure 3 that spot-like particles of SiO_2_ were formed. The particle sizes were between 20 and 80 nm, all less than 100 nm. The decrease in distance between particles increases with the number of particles. When the amount of nano-SiO_2_ was 5%, a slight agglomeration was observed in the coating, which may be due to the increase in the number of nano-SiO_2_ particles, the corresponding increase in the viscosity of the system, the decrease in the distance between the SiO_2_ nanoparticles, and the increase in interactions.

### 3.5. XRD Analysis of Composite Coatings

**Figure 4:**
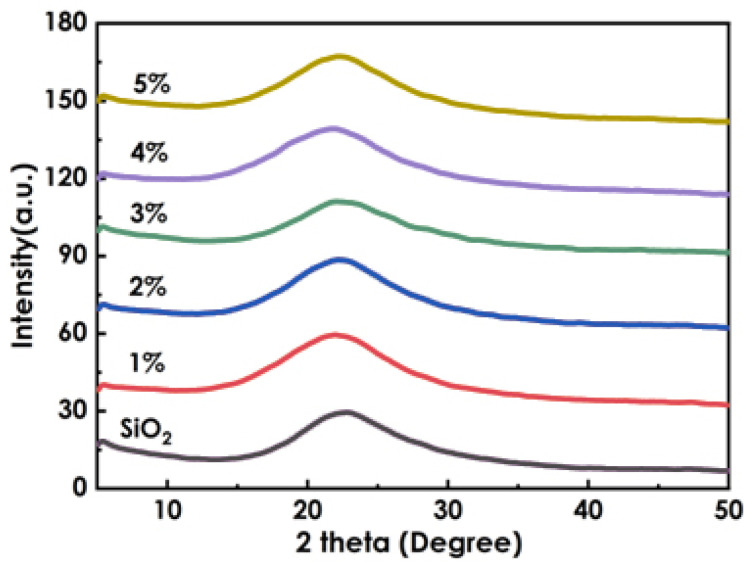
XRD patterns of coatings with different amounts of SiO_2_.

Figure 4 shows the standard spectrum of SiO_2_ and the spectra for different added amounts. The microstructures of the untreated SiO_2_ and composite coatings were compared using XRD. As shown in Figure 4, no sharp test peak was observed near 22.32°, whereas a steamed bread peak with a width close to the height was observed. This observation indicates extremely low crystallinity and an almost amorphous microstructure. The absorption peak of SiO_2_ in the composite coating shifts slightly to the left. This offset may have been caused by residual stress within the material. Compared with the characteristic peak standard card of SiO_2_ (JCPDS29 0085), SiO_2_ mainly existed in an amorphous state before and after treatment.

### 3.6. Thermogravimetric Analysis of Composite Coatings

**Figure 5:**
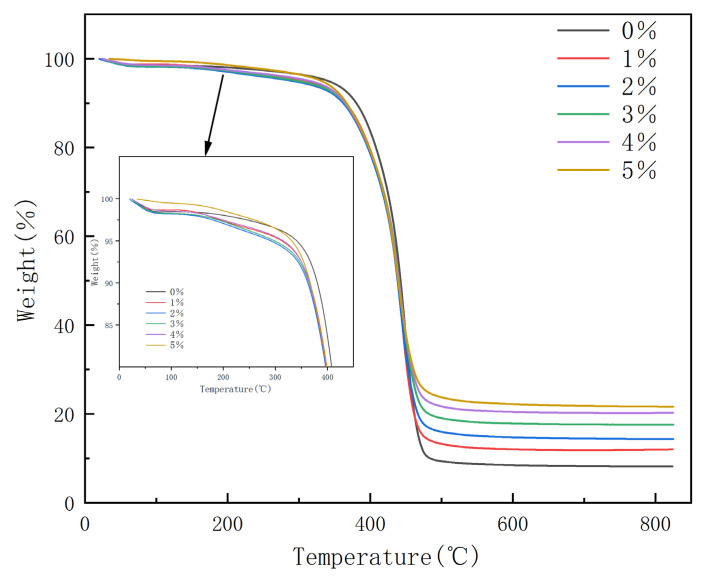
TG of coatings with different SiO_2_ contents.

**Figure 6:**
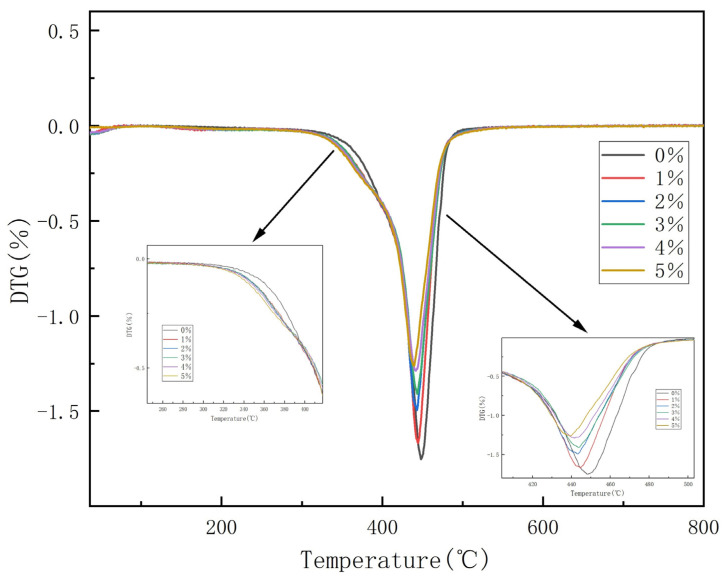
DTG of coatings with different SiO_2_ contents.

Figure 5 and Figure 6 show the TG and DTG graphs, respectively, for the coatings with varying SiO_2_ contents. As shown in Figure 5, the decomposition process of the coating at 0–800 °C can be divided into two stages, namely, 0–98 °C and 98–480 °C. According to Figure 6, the maximum weight loss peak of the coating was at approximately 400 °C. The first stage of weight loss was primarily due to the volatilization of residual solvents and free water in the system, with a weight loss rate of approximately 2%. The second stage of weight loss was due to the decomposition of organic matter, such as the resin and monomers, after UV curing. The figures indicate that the thermal stability of the coatings that contained 5% SiO_2_ is superior to that of the other coatings. The superior stability can be ascribed to the Si–O–Si structure of the coatings, which is expected to hinder the decomposition of the polymer. With the increase in nano-SiO_2_, the thermal stability is increased. This may be attributable to the interrelation between inorganic and organic elements.

### 3.7. Wear Resistance Test of Composite Coatings

**Figure 7:**
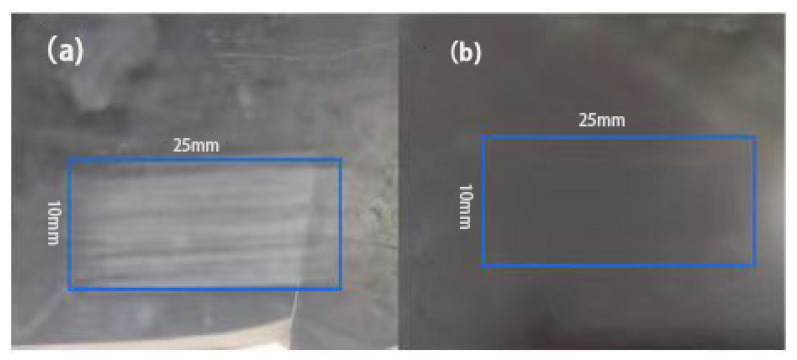
Images of wear damage seen during resistance testing. (**a**) the appearance of the coating without SiO_2_; (**b**) the appearance of the coating 3% nano-SiO_2_.

Figure 7a illustrates the appearance of the coating film without nano-SiO_2_ powder after 500 grinding cycles under a load of 1 kg, indicating serious wear damage. Figure 7b illustrates the appearance of the coating film with the addition of 3% nano-SiO_2_ powder after 2000 grinding cycles under a 1 kg load. This illustration clearly indicates an absence of visible scratches, only slight damage, and an intact substrate. This comparison demonstrates that the addition of nano-SiO_2_ greatly improved the wear resistance of the coating.

### 3.8. Contact Angle Test of Composite Coatings

**Figure 8:**
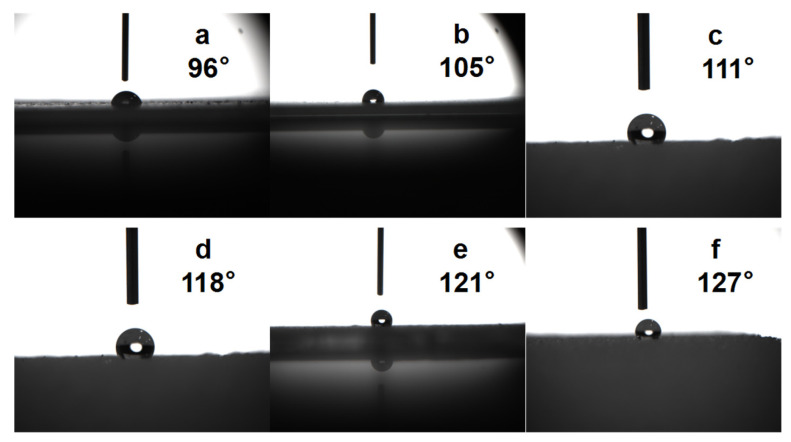
Contact angle tests of (**a**) organic coatings and composite coatings with (**b**) 1% SiO_2_, (**c**) 2% SiO_2_, (**d**) 3% SiO_2_, (**e**) 4% SiO_2_, and (**f**) 5% SiO_2_.

**Figure 9:**
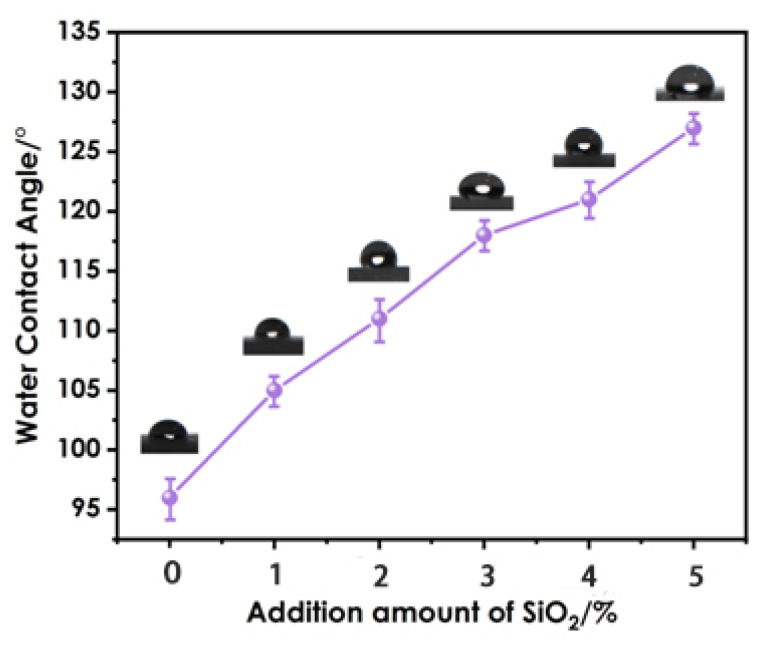
Variation diagram of coating contact angle for coatings with different SiO_2_ contents.

The contact angle of water on a material is an important parameter of the wettability of hydrophobic surfaces. Figure 8 and Figure 9 illustrate the effect of the nano-SiO_2_ content on the contact angle. The static contact angle of the measured surface of the organic coating devoid of nano-SiO_2_ was 96°, whereas the addition of nano-SiO_2_ at amounts of 1, 2, 3, 4 and 5% resulted in an increase in the static contact angles of the coatings to 105°, 111°, 118°, 121°, and 127°, respectively. Our findings demonstrate that the addition of nano-SiO_2_ particles provides excellent hydrophobicity to the coatings. This increase in contact angles is attributable to an increased roughness of the coating surface caused by an increased nano-SiO_2_ content. According to the wettability theory, the actual contact area between water droplets and the treated rough surface changes, leading to an increase in the solid–gas contact surface that results in an increase in the contact angle.

### 3.9. Evaluation of Haze and Mechanical Properties of Composite Coatings

**Figure 10:**
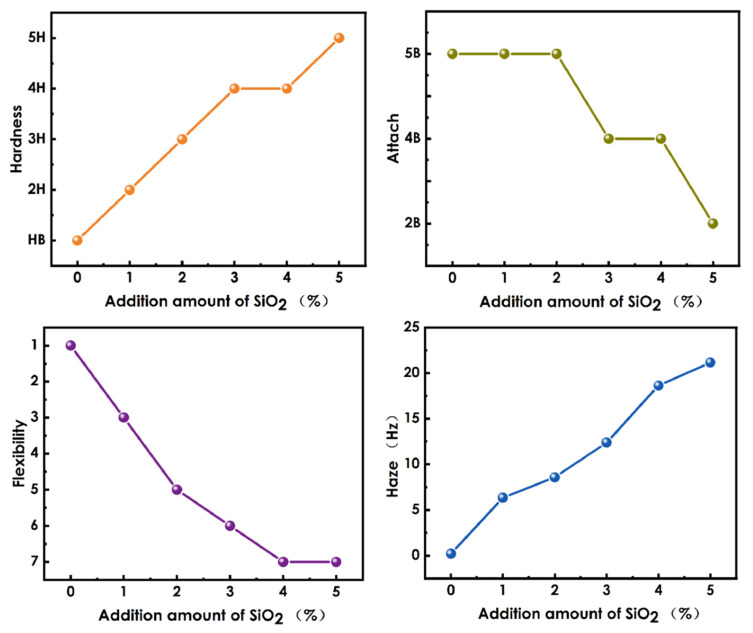
Graphs illustrating the effect of varying nano-SiO_2_ contents on the coating properties.

A total of 75 parts of B-619W, 10 parts of HDDA, 10 parts of DPHA, and 5 parts of 184 were weighed and mixed to an even consistency. Subsequently, 1–5% SiO_2_ and an appropriate amount of Afcona 4071 were added and mixed to achieve an even consistency. The mixture was ground for 30 min in a sand mill. The ground coating was diluted to a 40% solid content and then coated onto a 1.88 mm thick pet substrate using a 20 # wire rod. A performance test was then conducted on the cured film coating. As shown in Figure 10, an increase in SiO_2_ content improved the hardness of the film coatings significantly, with a corresponding increase in haze, whereas the flexibility and adhesion of the coatings decreased. At a nano-SiO_2_ content of 5%, the hardness of the coating reached 5H; however, the adhesion of the film was only 2B, and the flexibility was 1. The overall performance of the coating was optimal at a nano-SiO_2_ content of 3%. With this nano-SiO_2_ content, the coating exhibited good hardness, flexibility, and adhesion. The hardness reached 4H, the adhesion was 4B, the flexibility was 6, and the coating haze was 12.38 HZ.

## 4. Conclusions

In this study, nano-SiO_2_ particles were modified via high-energy ball milling and subsequently dispersed in ethyl acetate. The nano-SiO_2_ dispersions, containing nano-SiO_2_ particles with an average size of 64 nm, were cured in a UV-coating system. FTIR and XRD were used to evaluate the structure of the composite coatings. SEM analyses indicated that the modified SiO_2_ particles were evenly dispersed but exhibited light agglomeration throughout the coatings. Assessments of the mechanical and optical properties and thermal stabilities demonstrated that a 3% nano-SiO_2_ content provided an optimal composite coating, with hardness, adhesion, and flexibility reaching 4H, 4B, and 6. Under a load of 1 kg, which was applied over 2000 times, the coating haze reached 12.38 HZ, and the contact angle was 118°. The thermal stability of the composite coating was superior to that of the organic coatings. These findings confirm the successful preparation of a composite coating with excellent hardness and wear resistance, as well as a high contact angle. It has potential applications as a protective, hydrophobic, and wear-resistant coating.

## Figures and Tables

**Table 1:** Formulations with different monomer ratios.

	B-619W	HDDA	DPHA	184
Formula 1	80	15	0	5
Formula 2	80	10	5	5
Formula 3	80	7.5	7.5	5
Formula 4	80	5	10	5
Formula 5	80	0	15	5

**Table 2:** Performance of coatings with different monomer ratios.

	Hardness	Adhesion	Flexibility
Formula 1	H/500 g	5B	3
Formula 2	2H/500 g	4B	4
Formula 3	2H/750 g	4B	6
Formula 4	2H/1000 g	HB	7
Formula 5	3H/500 g	0B	8

**Table 3:** Evaluation of different resin ratios.

	B-619W	HDDA	DPHA	184
Formula 6	70	12.5	12.5	5
Formula 7	75	10	10	5
Formula 8	80	7.5	7.5	5
Formula 9	85	5	5	5
Formula 10	90	2.5	2.5	5

**Table 4:** Performance of coatings with different resin ratios.

	Hardness	Adhesion	Flexibility
Formula 6	2H/500 g	5B	8
Formula 7	HB/750 g	5B	1
Formula 8	2H/750 g	4B	6
Formula 9	H/1000 g	3B	6
Formula 10	H/750 g	2B	4

## Data Availability

Data are contained within the article.
